# Editorial

**DOI:** 10.1155/S1463924683000012

**Published:** 1983

**Authors:** Peter B. Stockwell


Journal of Automatic Chemistry, Volume 5, Number 1 (January–March 1983), pages 1–3

### Editorial

### Are we making the best use of laboratory computer systems?

In this first issue of the 1983 volume of the *Journal of Automatic Chemistry* we can look back on a very successful first year with our new publisher, Taylor & Francis Ltd. Their experience in scientific publishing has been of considerable value to me and the editorial team. We now have a well-produced, accepted and well-presented product. In 1983 we hope to build on this foundation and increase the readership still further. In some senses 1982 was a transition year. Many people will have had subscriptions based on a four-issue basis rather than an annual basis. In 1982 this has been rationalized and all subscriptions are now on an annual basis.

In 1982 much of our editorial matter centred on the use of computers in the laboratory, be it clinical or industrial. The application of computers is a very difficult subject to deal with adequately since the range of experience within the readership is so diverse. What is commonplace for one group is beyond the experience of many others. However, we have attempted in our editorial policy to cater for this and to present detailed examples of practical applications. We will continue to pursue this policy. Should any reader have specific problems relating to interfacing and developing software, we will endeavour to answer such questions in our pages. Within the editorial team we have a wealth of practical experience in applications and in the teaching aspects involved.

Despite the fact that computers and microcomputers are becoming commonplace in our lives, we continue to hear stories of difficulties being experienced implementing computer systems or, indeed, abject failures of systems installed to meet specific needs. In a guest editorial in the *Journal of Clinical Laboratory Automation* (Vol. 2, No. 6) Professor D. M. Block summarizes a series of failures, for a variety of reasons, and warns us to be extremely wary of the so-called 'expert computer consultant'. He tells us 'Be wary of those who teach the art and science of laboratory computing' and, when we are told something by these experts, to question upon what actual personal experience of laboratory computing they are basing their opinions. These are wise words indeed. However, what Professor Block does not suggest is an alternative approach which does not cause so many problems.

Having been through the various stages of defining, specifying, and implementing a laboratory system, I can perhaps add some points which can be of value. Firstly, the various computer experts may well be experts in the use of computers, but it is unlikely that they will have any experience of the particular laboratory for which they are consulting, nor will they have any concept of the staff structure and qualifications of the staff. Secondly, it is vitally important that the person who specifies the final system and justifies its choice and purchase must also be the person who has the responsibility to install the system. Choices made by committees without the need to make the system work rarely come to a good conclusive ending.

The main problem in any system study, be it completely computer or any automation instrumentation, is to find a complete and precise specification of the requirements. This should outline the needs of the organization, its staff, and its customers jointly. At this stage it should not make any attempt to suggest how to solve the problem outlined in the specification. In order to get to this stage correctly, the staff involved must make a real attempt to understand what can and cannot be achieved using computers. Exactly how difficult something is to achieve by computer also has to be realized. Specialized software is an extremely expensive commodity. A detailed specification must also make allowance for one extremely important variable: chance. Specifications often relate to a period of time—implementation and procurement themselves also take time—and the result of this is often that the system is subsequently installed to meet a rather different requirement. However, the difficulty in any specification is to be able to cover as many of these eventualities as possible. A good system, properly specified and installed, will meet many of the precise aims set out in the specification as originally outlined, but in addition will almost always provide additional benefits as a bonus. Without the full involvement and commitment of the users and scientists from the outset, and throughout the many stages involved in the introduction of computer systems into the laboratory, it will be difficult to make full use of the computer.

Hopefully, the many papers which we publish will be of direct help to new computer users. It is, however, a great pity that many of the pitfalls involved are not highlighted in the literature so that new users do not have to re-invent the wheel each time. Computers offer many advantages if properly used—it is correct to be wary of them but, given a good knowledge of their uses, advantages, and disadvantages, a lot can be gained by everyone in the laboratory situation. The scientist must help to identify the needs correctly so that the computers are used wisely, both within laboratories and by instrument companies alike.

Best wishes for successful computing in 1983.

**Peter B. Stockwell**
*Editor*

### Comments

### Flow-injection analysis—The end of the beginning? Segmented-flow analysis—The beginning of the end?

Although several reviews of flow-injection analysis (FIA) have been published [1–4], it is clear from recent remarks made in this *Journal* by Holy [5] that the technique is still not properly understood. We should therefore like to take this opportunity of restating some of the essential features of FIA and of replying to some of the criticisms raised in that paper. We have an advantage over Holy in that we are not committed to any particular mode of automatic analysis. Proponents of FIA may draw comfort from the fact that the Technicon Corporation originally adopted a similar stance with regard to discrete analysis but now actually market two discrete analysers.

As Holy points out, analytical chemists have been pumping

